# From Waste to Resource: Reframing End‐of‐Life Materials for a Circular Future: Within MSE Symposium Sustainable Reprocessing of End‐of‐Life Materials and Side Streams

**DOI:** 10.1002/gch2.70116

**Published:** 2026-05-26

**Authors:** Matic Jovičević‐Klug, Denys Villa Gomez, Carlo Alberto Brondin, Patricia Jovičević‐Klug

The accelerating pace of industrialisation and consumption has led to an unprecedented accumulation of materials that have reached the end of their functional lifetime. These end‐of‐life materials, ranging from complex alloys and composites to functional inorganic systems, represent a growing environmental burden, as well as profound systemic inefficiency and material loss. Societies across the globe are grappling with mounting volumes of waste that strain local economies, challenge existing infrastructure, and contribute to environmental degradation. Yet within this challenge lies a transformative opportunity: to reconceptualise waste as a valuable resource of materials in the form of elements and compounds [1, 2].

The limited recyclability and reusability of many advanced materials remain key challenges. Modern products are typically designed with performance in mind rather than circularity, incorporating complex blends of elements and compounds that are challenging to separate and recover. Consequently, significant quantities of valuable materials are downgraded, disposed of in landfills, or lost entirely. The linear “take–make–dispose” model is becoming increasingly incompatible with the realities of resource scarcity, environmental constraints, and geopolitical pressures surrounding critical raw materials (CRMs) [3, 4]. The growing demand for CRMs, which are essential for technologies such as renewable energy systems, digital infrastructure, and advanced manufacturing, has intensified concerns about supply security. Many of these materials are geologically scarce and geographically concentrated, making them environmentally costly to extract. At the same time, however, significant quantities of these same elements are already present in end‐of‐life products and industrial residues. Therefore, unlocking this secondary resource base through efficient and sustainable recovery processes is an environmental and strategic necessity [1, 5].

Parallel to the challenge of post‐consumer waste is the issue of industrial side streams. Across sectors, production processes generate vast quantities of byproducts and residues that require careful management. Historically treated as waste requiring disposal or costly remediation, these side streams are increasingly being reconsidered as unconventional resource pools. Advances in analytical techniques and process engineering have revealed that many industrial wastes contain appreciable concentrations of technologically relevant elements and compounds. As primary ore grades decline and extraction costs rise, the incentive to valorize these secondary sources continues to grow [1, 6]. However, realising this potential requires overcoming significant technical, economic, and systemic barriers. The heterogeneous, contaminated, or dispersed nature of many waste streams complicates their collection and processing. Existing recycling technologies are often energy‐intensive, economically unviable on a large scale, or limited to specific applications with low overall reprocessing efficiency. Furthermore, regulatory frameworks and market structures often fail to keep pace with technological capabilities, hindering the adoption of innovative solutions. Addressing these challenges demands a holistic approach that integrates materials science, chemistry, environmental science, and policy development [3, 7, 8].

In this context, the concept of a circular economy offers a compelling framework. Rather than viewing materials as disposable, the circular approach seeks to maintain their value by repeatedly using, recovering, and regenerating them. This requires advances in recycling technologies, as well as a fundamental rethink of product design, supply chains, and consumption patterns. Designing materials and products for disassembly, recovery, and reuse can significantly increase their value at the end of their life and simplify recycling processes. Technological innovations will play a pivotal role in enabling this transition. Emerging approaches such as selective leaching, biohydrometallurgy, electrochemical recovery, and advanced separation techniques offer promising ways of extracting high‐value materials from complex waste streams. Digital tools, including artificial intelligence and data‐driven process optimisation, can further improve the efficiency, adaptability, and scalability of these processes. At the same time, interdisciplinary collaboration is essential to bridge the gap between laboratory‐scale breakthroughs and industrial implementation. It is equally important to develop integrated process chains that encompass the entire lifecycle of materials, from collection and pre‐treatment to recovery, purification, and reintegration into manufacturing. These systems must be technically robust, economically viable, and environmentally sustainable. Life cycle assessment and techno‐economic analysis are critical tools for evaluating these systems' performance and guiding decision‐making.

The *Global Challenges* collection titled “From Waste to Resource: Reframing End‐of‐Life Materials for a Circular Future” aims to highlight the latest developments and emerging trends in the sustainable processing of end‐of‐life materials and industrial waste. We invite contributions that explore novel approaches to the recycling of inorganic materials, including oxides, alloys, composites, and functional systems. Studies on the innovative reprocessing of industrial waste, the alternative use of residual materials, and disruptive concepts and technologies for extracting high‐value elements and CRMs are also welcome. Particular emphasis is placed on integrated circular process chains that enable the efficient recovery and reuse of materials throughout their lifecycle. Research into novel beneficiation techniques, scalable processing methods, and system‐level optimisation is critical to advancing the field and is therefore also welcome. Interdisciplinary perspectives that combine technical innovation with economic, environmental, and policy considerations are especially encouraged.

## Conflicts of Interest

The authors declare no conflicts of interest.

## Data Availability

Data sharing not applicable to this article as no datasets were generated or analyzed during the current study.
